# Supplementary material for: SARS-CoV-2 infection induces DNA damage, through CHK1 degradation and impaired 53BP1 recruitment, and cellular senescence
Source: Nat Cell Biol. 2023 Mar 9;25(4):550–64. doi: 10.1038/s41556-023-01096-x (PMC10104783; doi:10.1038/s41556-023-01096-x)
Supplement: Supplementary file 1 — Reporting Summary [file 41556_2023_1096_MOESM1_ESM.pdf]

nature portfolio

Corresponding author(s): Fabrizio d'Adda di Fagagna

Last updated by author(s): Jan 12, 2023

Reporting Summary

Nature Portfolio wishes to improve the reproducibility of the work that we publish. This form provides structure for consistency and transparency in reporting. For further information on Nature Portfolio policies, see our Editorial Policies and the Editorial Policy Checklist.

Statistics

For all statistical analyses, confirm that the following items are present in the figure legend, table legend, main text, or Methods section.

n/a	Confirmed	
☐	☒	The exact sample size (*n*) for each experimental group/condition, given as a discrete number and unit of measurement
☐	☒	A statement on whether measurements were taken from distinct samples or whether the same sample was measured repeatedly
☐	☒	The statistical test(s) used AND whether they are one- or two-sided<br>*Only common tests should be described solely by name; describe more complex techniques in the Methods section.*
☐	☒	A description of all covariates tested
☐	☒	A description of any assumptions or corrections, such as tests of normality and adjustment for multiple comparisons
☐	☒	A full description of the statistical parameters including central tendency (e.g. means) or other basic estimates (e.g. regression coefficient) AND variation (e.g. standard deviation) or associated estimates of uncertainty (e.g. confidence intervals)
☐	☒	For null hypothesis testing, the test statistic (e.g. *F*, *t*, *r*) with confidence intervals, effect sizes, degrees of freedom and *P* value noted<br>*Give P values as exact values whenever suitable.*
☒	☐	For Bayesian analysis, information on the choice of priors and Markov chain Monte Carlo settings
☒	☐	For hierarchical and complex designs, identification of the appropriate level for tests and full reporting of outcomes
☒	☐	Estimates of effect sizes (e.g. Cohen's *d*, Pearson's *r*), indicating how they were calculated

Our web collection on statistics for biologists contains articles on many of the points above.

Software and code

Policy information about availability of computer code

Data collection	Bio-Rad Image Lab 6.1 was used for immunoblot data collection; images at widefield microscope were acquired with MetaVue software or with Zen 2.0 Software (Zeiss); images at confocal microscope were collected with Leica Application Suite X; images in micro-injection experiments were acquired with Volocity 6.4.0; BioPlex 200 system (Bio-Rad), powered by the Luminex xMAP technology was used for multiplex immunoassays; for flow cytometry studies, see the related section.
Data analysis	Bio-Rad Image Lab 6.1 was used for densitometric analysis in immunoblot experiments; CellProfiler 3.1.9 was used to measure DDR activation in immunofluorescence analyses and in micro-injection experiments; tail moment in comet assays was measured using CometScore 2.0; ImageJ 1.53a was used to quantify protein sub-cellular distribution in immunofluorescence experiments; GraphPad Prism 9.3.0 was used for statistics; quantitative analyses of IHC experiments were performed using the Nuclear v9 or Positive Pixel Count v9 ImageScope software (v12.3.2.8013, Leica Biosystems); combined ISH/IHC images were analyzed with HALO v3.5.3577.140 (Indica Labs); for flow cytometry studies, we used FlowJo 10.7.1 (BD Biosciences).

For manuscripts utilizing custom algorithms or software that are central to the research but not yet described in published literature, software must be made available to editors and reviewers. We strongly encourage code deposition in a community repository (e.g. GitHub). See the Nature Portfolio guidelines for submitting code & software for further information.

Data

Policy information about availability of data

All manuscripts must include a data availability statement. This statement should provide the following information, where applicable:

- Accession codes, unique identifiers, or web links for publicly available datasets
- A description of any restrictions on data availability
- For clinical datasets or third party data, please ensure that the statement adheres to our policy

No datasets were generated or analysed during the current study. All raw data associated to Figures in manuscript are provided as supplementary material or available upon reasonable request.

Human research participants

Policy information about studies involving human research participants and Sex and Gender in Research.

Reporting on sex and gender	Sex and gender analysis was not necessary for this study
Population characteristics	We analysed lung parenchyma from 17 COVID-19 patients and nasal mucosa from 18 COVID-19 patients. Detailed information on patient's characteristics and treatment are provided in (Bussani et al., 10.1016/j.ebiom.2020.103104). The same number of lung samples was analysed from non-COVID patients, who died with viral pneumonia of different etiologies. It was not possible to analyze COVID-19-negative mucosae, as the harvesting procedure of the nasal mucosa is highly invasive and it destroys the appearance of the face; thus it was justified only during the pandemic in COVID-19 patients.
Recruitment	Patients were not recruited specifically for this study. All samples were previously collected from autopsy cases of COVID-19 and non-COVID patients, and then grouped according to their positivity for SARS-CoV-2.
Ethics oversight	This study was approved by the competent Joint Ethics Committee of the Regione Friuli Venezia Giulia, Italy. All patients provided their written informed consent to the use of their samples for research purposes at the time of hospital admission.

Note that full information on the approval of the study protocol must also be provided in the manuscript.

Field-specific reporting

Please select the one below that is the best fit for your research. If you are not sure, read the appropriate sections before making your selection.

☒ Life sciences ☐ Behavioural & social sciences ☐ Ecological, evolutionary & environmental sciences

For a reference copy of the document with all sections, see nature.com/documents/nr-reporting-summary-flat.pdf

Life sciences study design

All studies must disclose on these points even when the disclosure is negative.

Sample size	We did not use any criteria to determine the sample size. As much data as possible was collected depending on the nature of the experiments or in order to have statistical analysis
Data exclusions	Throughout the manuscript no data was excluded. Only in rare occasions, individual values were removed following unbiased criteria of outlier identification using Prism 9 software. In addition, in the case of IHC staining in infected mouse lungs, we excluded the tissue sections that show low rates of SARS-CoV-2 infection.
Replication	For all the experiments at least 3 independent replicates were performed unless differently stated in the figure legends.
Randomization	For in vivo experiments in mice, animals were randomized to the experimental groups. For in vitro experiments, wells were randomly assigned into each group and all cells were analysed equally.
Blinding	No blinding method was applied, as we used unbiased software for data collection and analysis.

Reporting for specific materials, systems and methods

We require information from authors about some types of materials, experimental systems and methods used in many studies. Here, indicate whether each material, system or method listed is relevant to your study. If you are not sure if a list item applies to your research, read the appropriate section before selecting a response.

Materials & experimental systems

n/a	Involved in the study	
☐	☒	Antibodies
☐	☒	Eukaryotic cell lines
☒	☐	Palaeontology and archaeology
☐	☒	Animals and other organisms
☒	☐	Clinical data
☒	☐	Dual use research of concern

Methods

n/a	Involved in the study	
☒	☐	ChIP-seq
☐	☒	Flow cytometry
☒	☐	MRI-based neuroimaging

Antibodies

Antibodies used

gH2AX (Ser139) Abcam ab11174
gH2AX (Ser139) Millipore 05-636
53BP1 Bethyl A303-906A
53BP1 Novus NB100-304
ACE2 Abcam ab15348
ATM Abcam ab32420
ATR Santa Cruz sc-1887
Beclin 1 Bethyl A302-566A-T
Beta-actin Sigma-Aldrich A2228
BrdU BDbioscience 347580
CD68 Abcam ab125212
CDT1 Cell Signaling #8064
cGAS Cell Signaling #15102
CHK1 Novus NB100-46
CHK1 (2G1D5) Cell Signaling #2360
CHK1 (ST57-09) ThermoFisher MA532180
CHK2 Millipore 05-649
Cleaved Caspase-3 (Asp 175) Cell Signaling 9661
DNA-PK Abcam ab32566
HA-tag Abcam ab236632
Histone H3 Abcam ab10799
Human Cytokeratin 8/18 (EP17/EP30) Dako M3652
ISceI (FL-86) Santa Cruz sc-98269
KAP1 Abcam ab10484
LC3B Sigma-Aldrich L7543
p16 Abcam ab51243
p21 Abcam ab188224
P21 Cell Signaling #2946
P38 MAPK Cell Signaling #9212
P53 Abcam ab1101
p62 Abcam ab240635
pATM (Ser1981) Rockland 200-301-400
pATR (T1989) Abcam ab223258
pCHK1 (S317) Cell Signaling #2344
pCHK2 (Thr68) Cell Signaling #2661
PCNA Bio Rad MCA1558
pDNA-PK (Ser2056) (EPR5670) Abcam ab124918
pKAP1 (S824) Bethyl A300-767A
pP38 MAPK (Thr180/Tyr182) Cell Signaling #9211
pP53 (Ser15) Cell Signaling #9284
proSP-C Abcam ab3786
pRPA (S4/S8) Bethyl A300-245A
pSTAT1 (Ser 727) Cell Signaling #9177
pSTAT1 (Tyr 701) (58D6) Cell Signaling #9167
RPA Calbiochem NA18-100UG
RRM1 Santa Cruz sc-11733
RRM2 Novus NBP1-31661
RRM2 Santa Cruz sc-10844
SARS-CoV2 nucleocapsid Sino Biological 40588-T62
SARS-CoV2 nucleocapsid Sino Biological 40143-R019
SARS-CoV2 nucleocapsid (1A6) ThermoFisher MA5-35941
STAT1 (9H2) Cell Signaling #9176
Strep-tag II epitope Qiagen 34850
Tubulin Sigma-Aldrich T5168
Ubiquitin (P4D1) Santa Cruz sc-8017
Vinculin Sigma-Aldrich V9131
Cy3 D/M Jackson 715-165-150
Cy3 D/R Jackson 711-165-152
Cy3 D/G Jackson 705-165-147

	A488 D/M ThermoFisher A21202<br>A488 D/R ThermoFisher A21206<br>A488 D/G ThermoFisher A11055<br>A647 D/M ThermoFisher A31571<br>A647 D/R ThermoFisher A31573<br>A647 D/G ThermoFisher A21447
Validation	All antibodies were validated by the manufacturer and were previously used in peer reviewed works. Methods of validation and references to published application for all antibodies are all present into manufacturer dedicated website page of each indicated product.

Eukaryotic cell lines

Policy information about cell lines and Sex and Gender in Research

Cell line source(s)	Vero E6 cells (ATCC-1586); human hepatocarcinoma Huh7 cells (JCRB0403, JCRB cell bank of Okayama University) were kindly provided by Ralf Bartenschlager, University of Heidelberg, Germany; lung adenocarcinoma Calu-3 (ATCC HTB-55); U2OS 53BP1-GFP (Bekker-Jensen et al. 2005); U2OS EJ5-GFP (Gunn & Stark 2012); NIH2/4 (Soutoglou et al. 2007)
Authentication	Cell lines were authenticated by STR profiling (GenePrint system, Promega)
Mycoplasma contamination	All cell lines were tested negative for mycoplasma
Commonly misidentified lines<br>(See ICLAC register)	No commonly misidentified lines were used

Animals and other research organisms

Policy information about studies involving animals; ARRIVE guidelines recommended for reporting animal research, and Sex and Gender in Research

Laboratory animals	Ten B6.Cg-Tg(K18-ACE2)2Prlmn/J mice (M. musculus; two 13-week-old females; two 11-week-old males, four 10-week-old males, one 8.5-week-old male and one 8.5-week-old female); four C57BL/6 J mice (M. musculus; two 11-month-old males and two 10-week-old males).
Wild animals	This study did not involve wild animals.
Reporting on sex	Sex analysis was not necessary for this study.
Field-collected samples	This study did not involve samples collected from the field.
Ethics oversight	Experiments involving animals have been carried out in accordance with the Italian Laws (D.lgs. 26/2014), which enforce Directive 2010/63/EU (Directive 2010/63/EU of the European Parliament and of the Council of 22 September 2010 on the protection of animals used for scientific purposes). Accordingly, the project has been authorized by the Italian Competent Authority (Ministry of Health).

Note that full information on the approval of the study protocol must also be provided in the manuscript.

Flow Cytometry

Plots

Confirm that:

☒ The axis labels state the marker and fluorochrome used (e.g. CD4-FITC).

☒ The axis scales are clearly visible. Include numbers along axes only for bottom left plot of group (a 'group' is an analysis of identical markers).

☒ All plots are contour plots with outliers or pseudocolor plots.

☒ A numerical value for number of cells or percentage (with statistics) is provided.

Methodology

Sample preparation	Experiments were carried out in cultured cells fixed first in formaldehyde 2% and then in 75% ethanol
Instrument	Samples were acquired with Attune NxT (Thermofisher)
Software	Analysis was carried out using FlowJo 10.7.1 (BD Biosciences)
Cell population abundance	30,000 events were analyzed for each sample in each individual experiment.
Gating strategy	Cell doublets were removed and living cells selection was based on forward and side scatter. Single cells were gated based on

Gating strategy

their SSC-A vs. FSC-A and SSC-A vs. SSC-H parameters. 561 nm laser and 695/40 filter were used for propidium iodide detection; 488 nm laser and 530/30 filter were used for BrdU and CHK1 detection.

☒ Tick this box to confirm that a figure exemplifying the gating strategy is provided in the Supplementary Information.
