# Supplementary figures and images for: SARS-CoV-2 infection induces DNA damage, through CHK1 degradation and impaired 53BP1 recruitment, and cellular senescence
Source: Nat Cell Biol. 2023 Mar 9;25(4):550–64. doi: 10.1038/s41556-023-01096-x (PMC10104783; doi:10.1038/s41556-023-01096-x)

## Slide 1
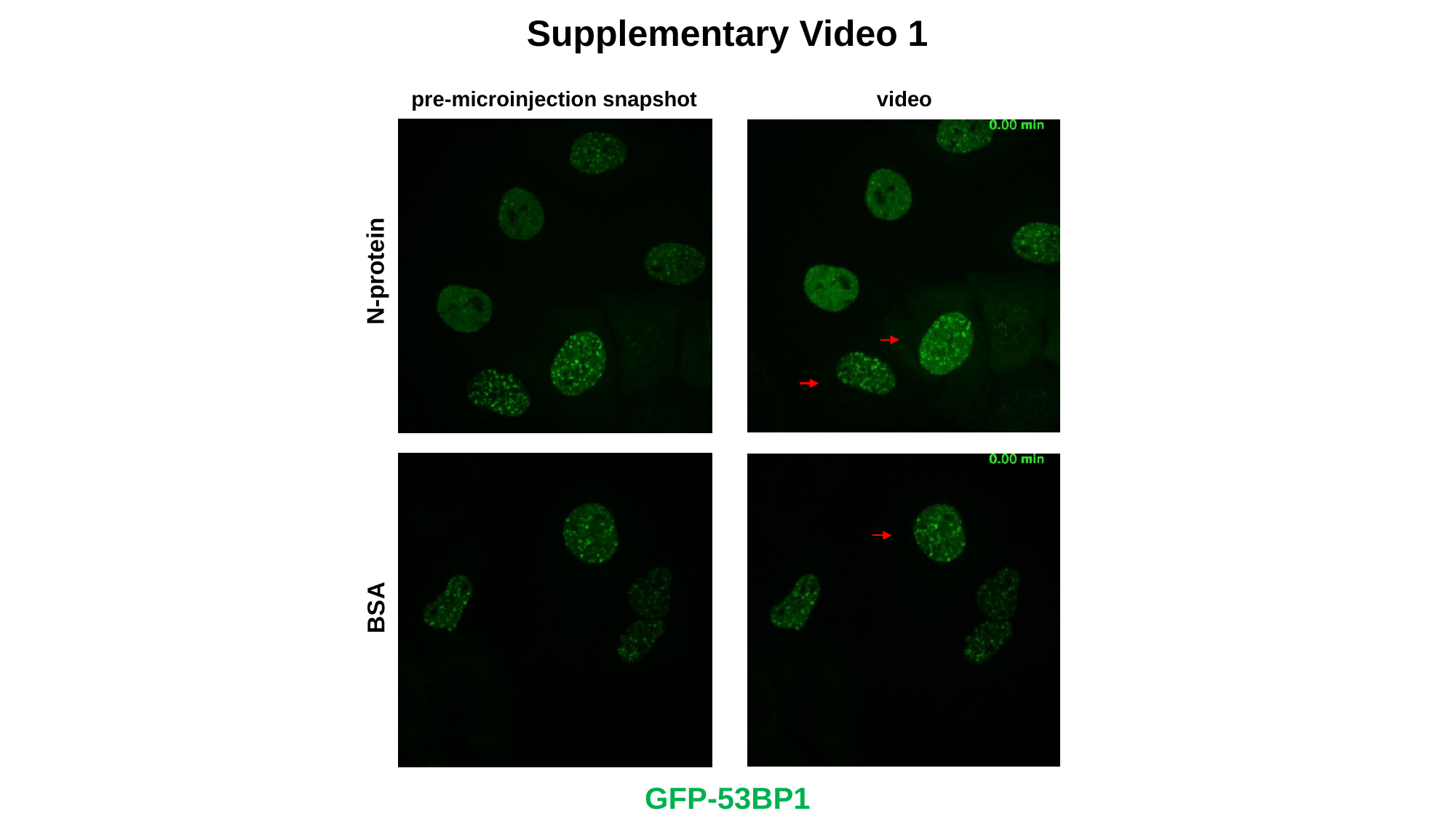

Supplementary Video 1
pre-microinjection snapshot
video
N-protein
BSA
GFP-53BP1

Supplement: Supplementary file 4 — Movie showing 53BP1-GFP foci over time upon micro-injection of 53BP1-GFP U2OS nuclei with recombinant SARS-CoV-2 N-protein or BSA as control. Arrows mark micro-injected cells. Images relative to pre-injected samples are also shown. The video was recorded at one frame per minute for 100 min total. The experiment was repeated three times with similar results. [file 41556_2023_1096_MOESM4_ESM.pptx]

Related to **Figure 1A**

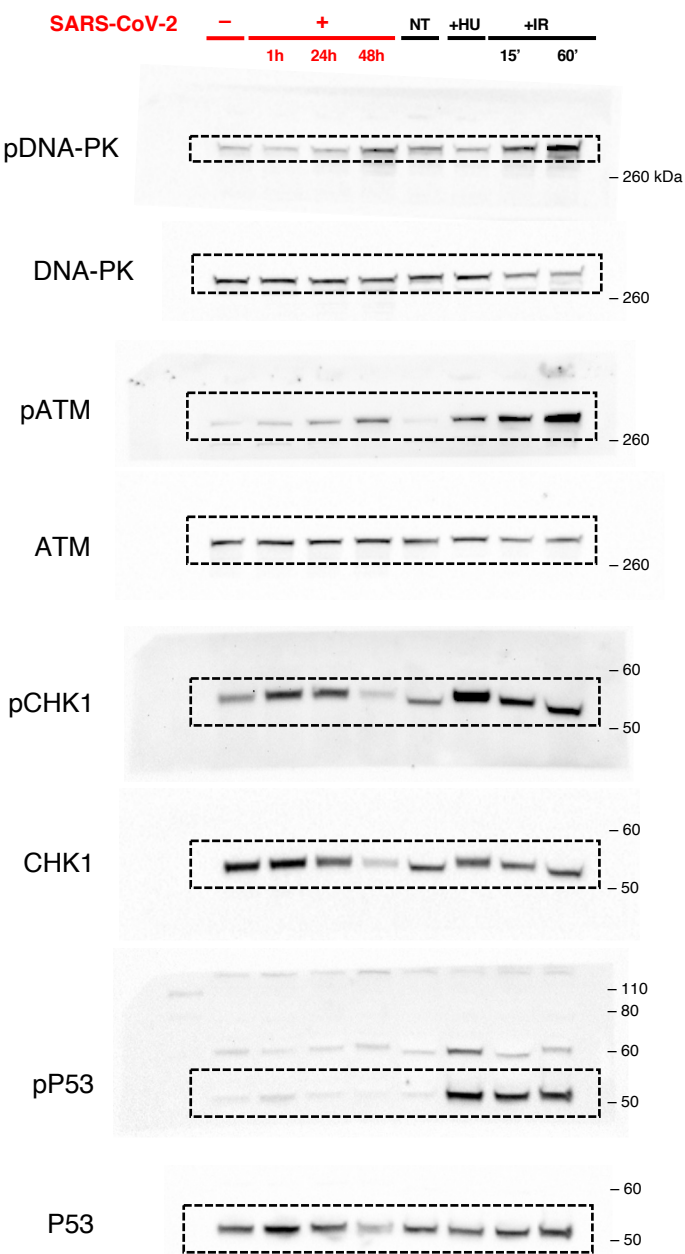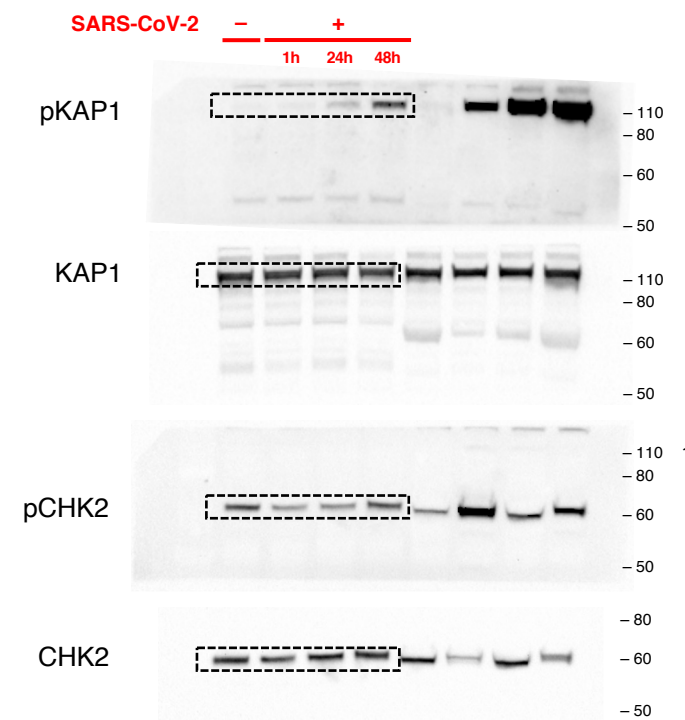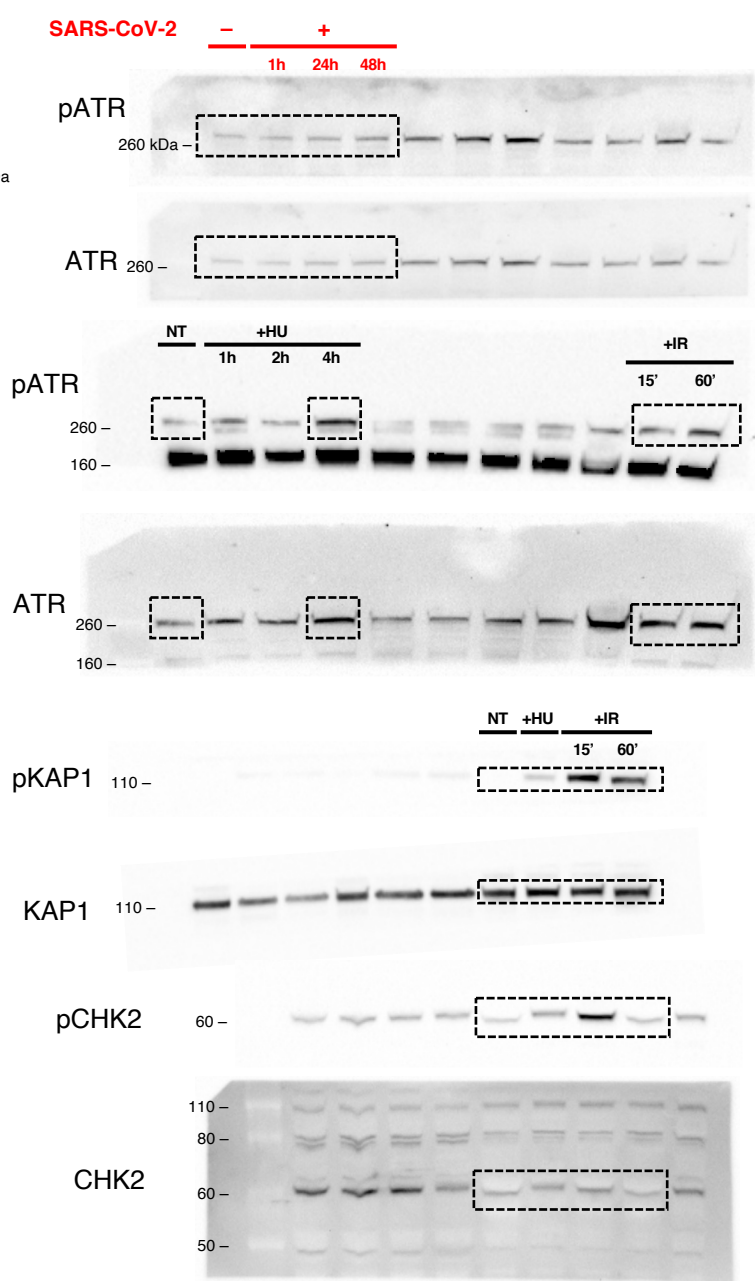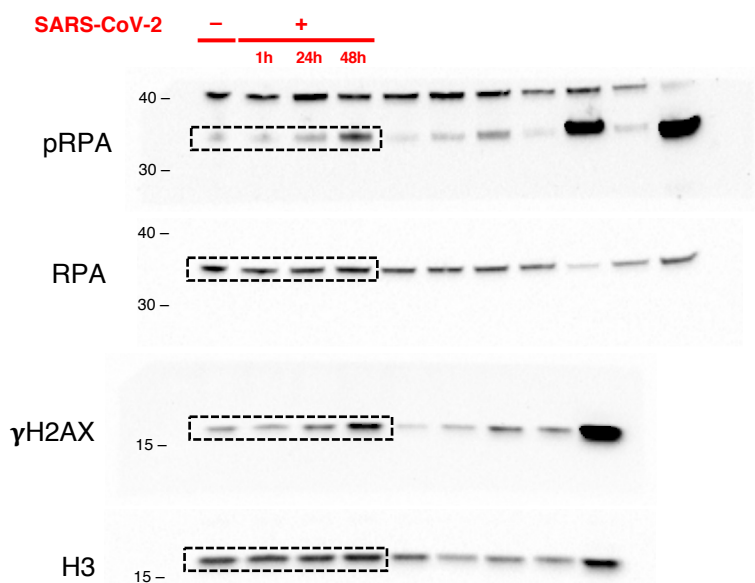

(continued)

(continued)

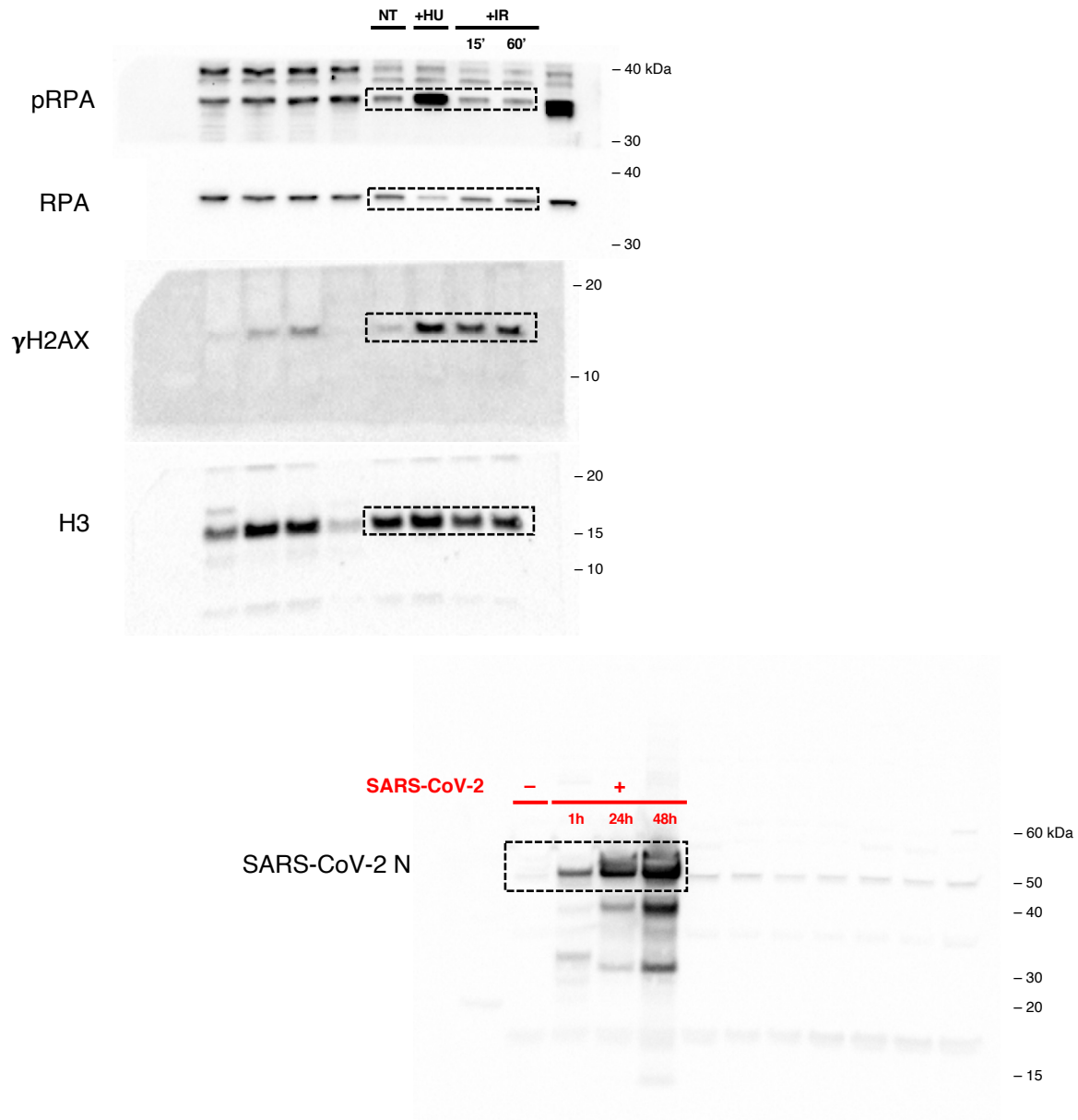

Supplement: Source Data Fig. 1 — Unprocessed western blots. [file 41556_2023_1096_MOESM6_ESM.pdf]

Related to **Figure 2A**

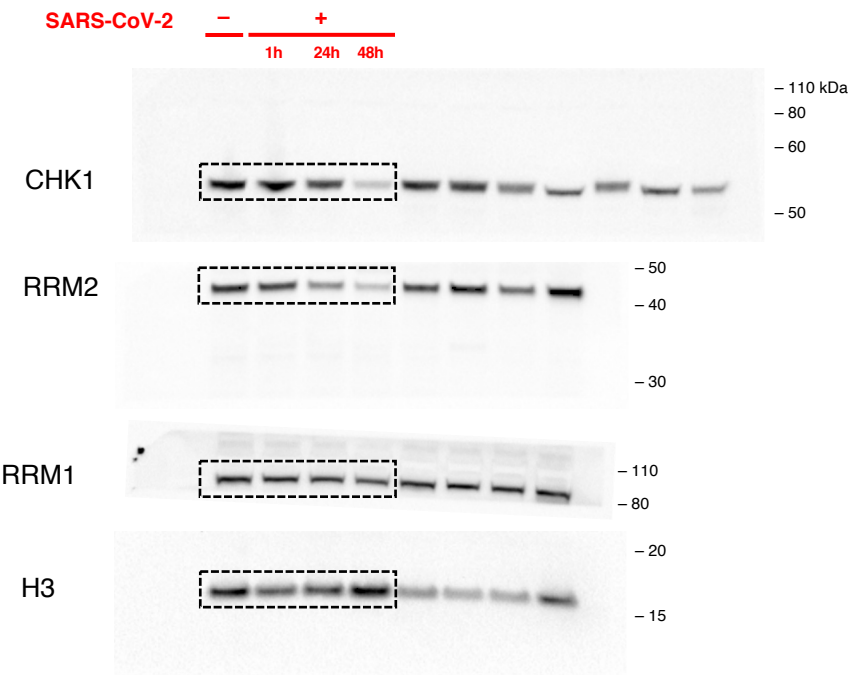

Supplement: Source Data Fig. 2 — Unprocessed western blots. [file 41556_2023_1096_MOESM8_ESM.pdf]

Related to **Figure 3C**

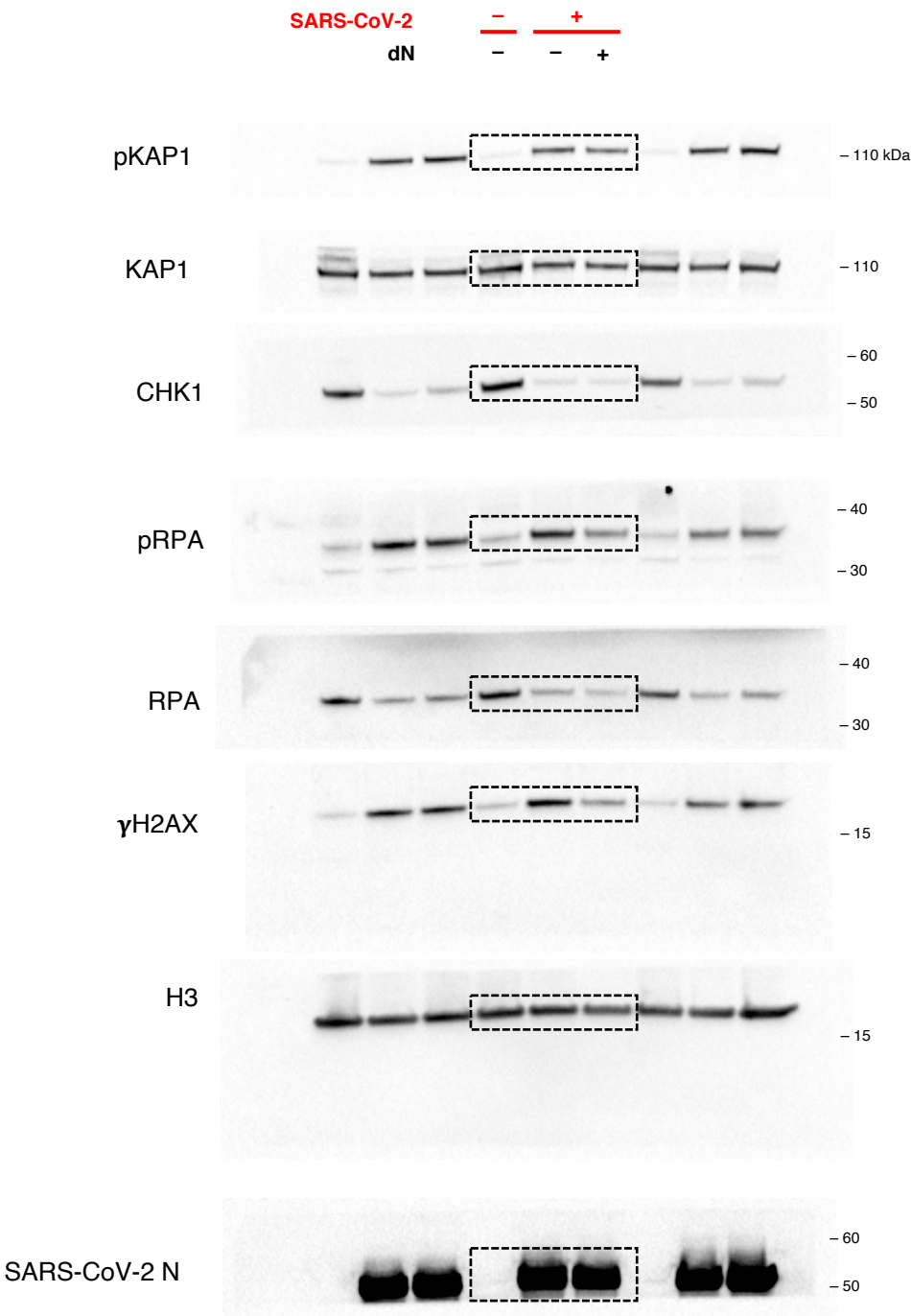

Supplement: Source Data Fig. 3 — Unprocessed western blots. [file 41556_2023_1096_MOESM10_ESM.pdf]

# Related to Figure 4C

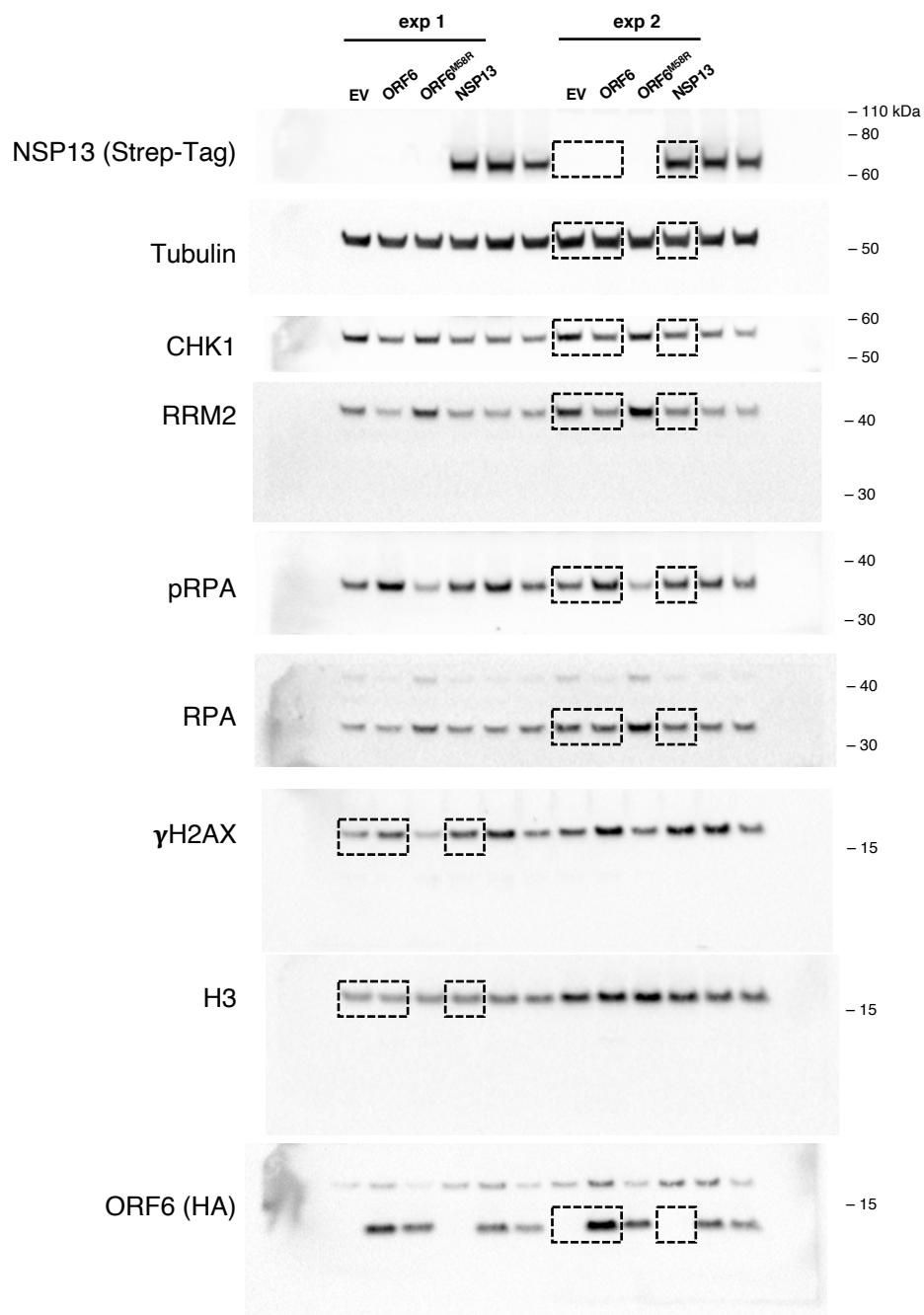

Supplement: Source Data Fig. 4 — Unprocessed western blots. [file 41556_2023_1096_MOESM12_ESM.pdf]

Related to **Figure 5C**

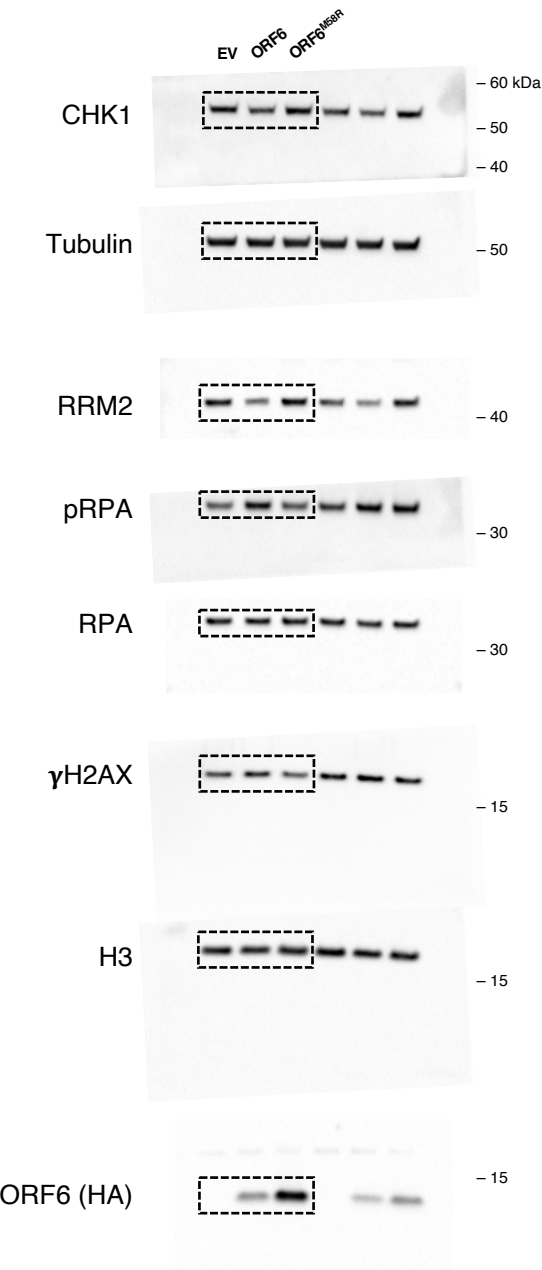

Related to **Figure 5G**

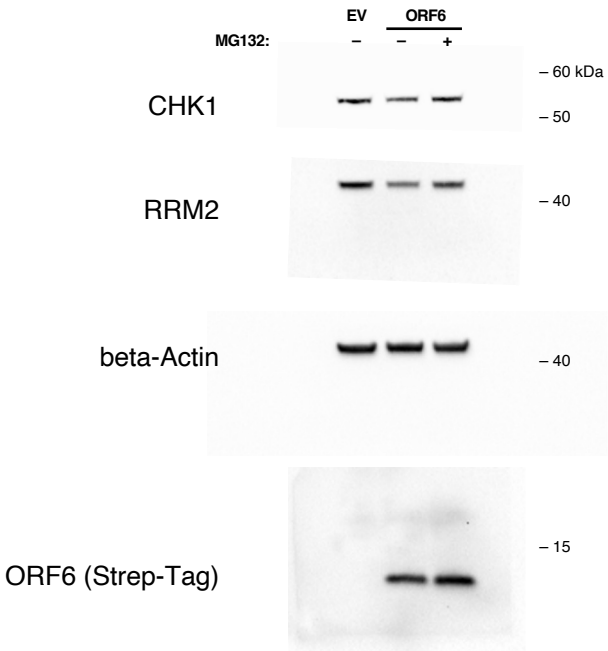

Related to **Figure 5I**

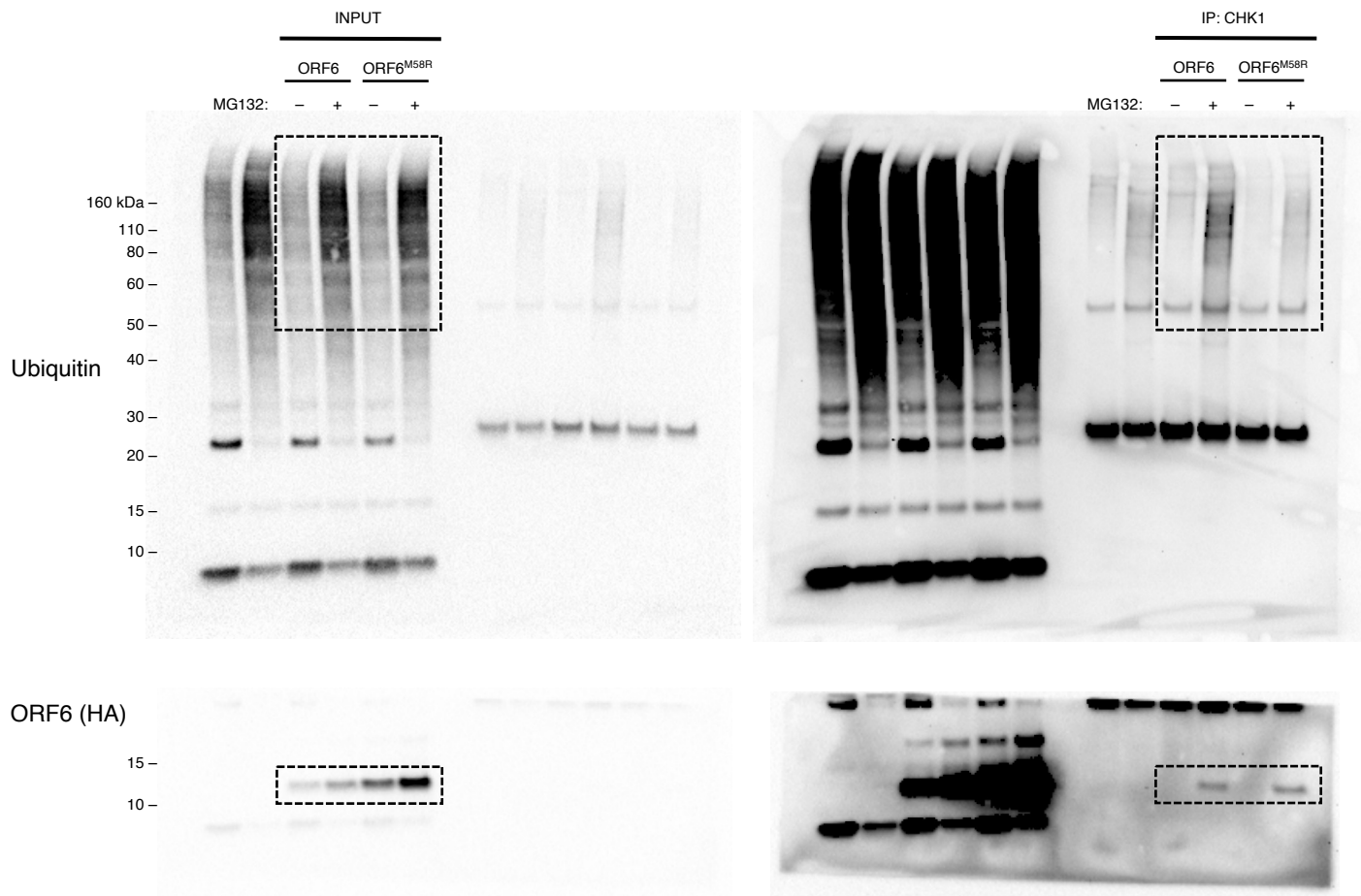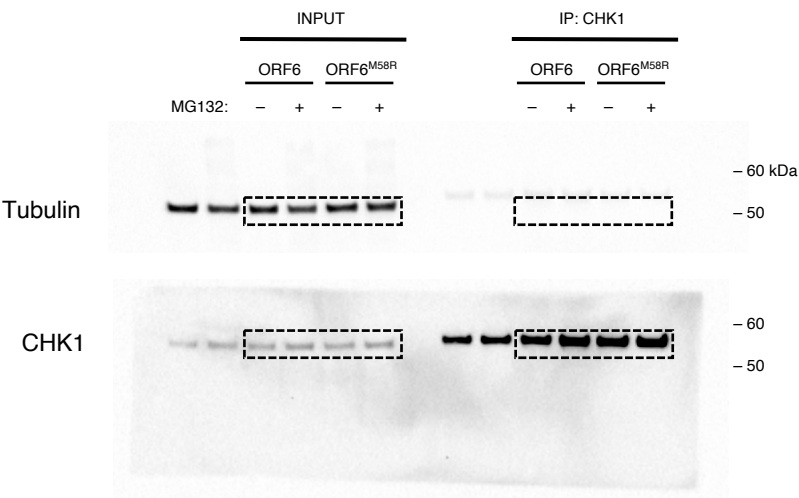

Supplement: Source Data Fig. 5 — Unprocessed western blots. [file 41556_2023_1096_MOESM14_ESM.pdf]

Related to **Extended Data Figure 2C**

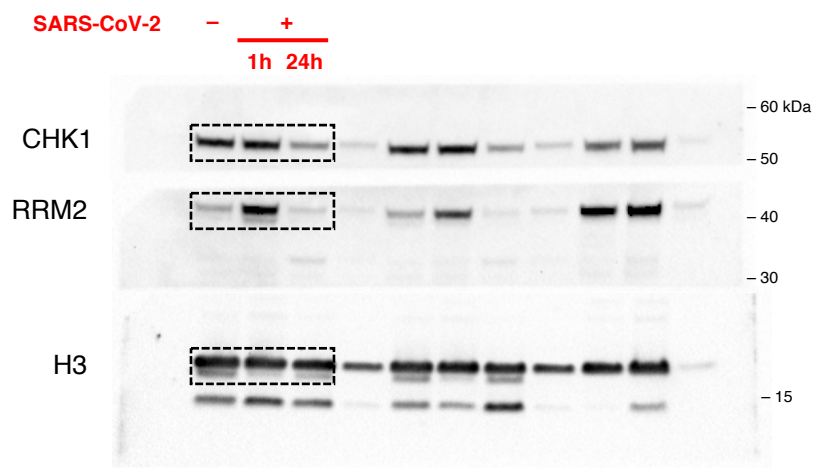

Related to **Extended Data Figure 2G**

**Huh7**

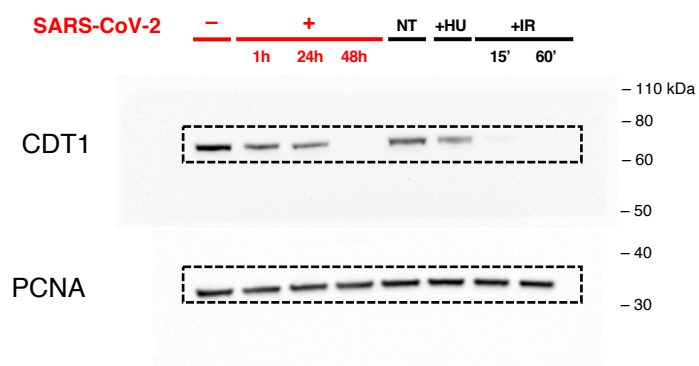

**Calu-3**

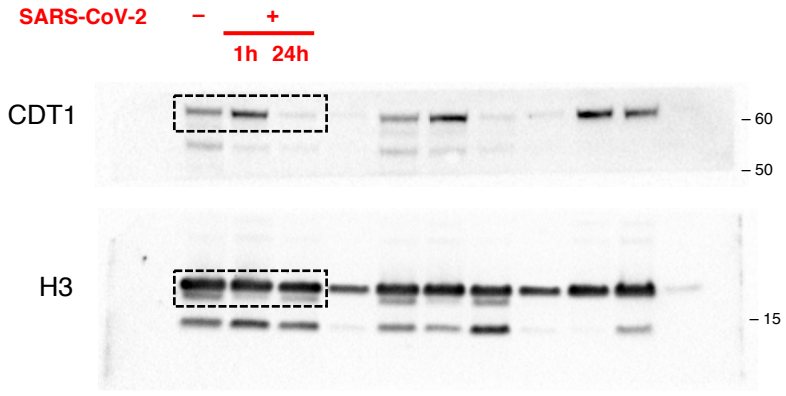

Supplement: Source Data Extended Data Fig. 2 — Unprocessed western blots. [file 41556_2023_1096_MOESM21_ESM.pdf]

Related to **Extended Data Figure 3E**

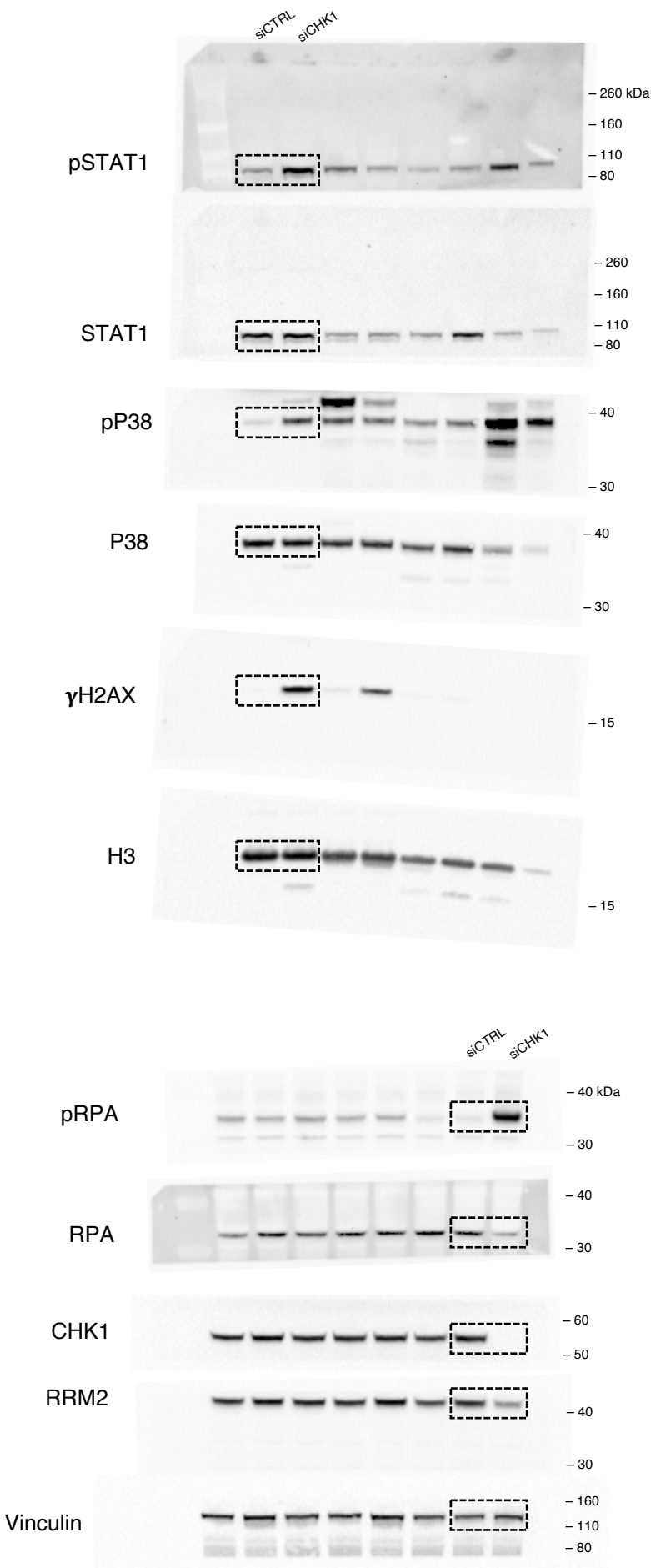

Supplement: Source Data Extended Data Fig. 3 — Unprocessed western blots. [file 41556_2023_1096_MOESM23_ESM.pdf]

Related to **Extended Data Figure 4A**

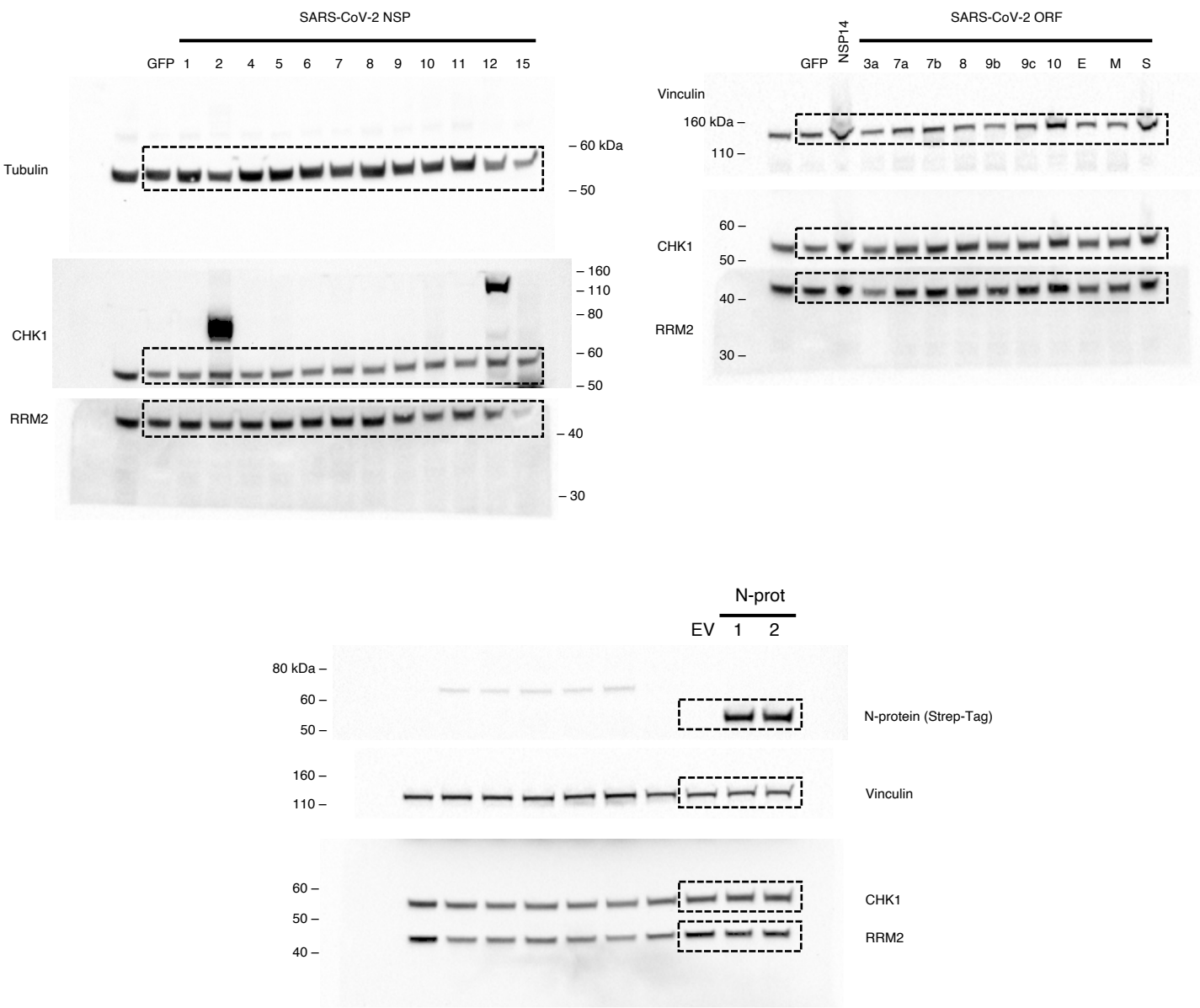

Supplement: Source Data Extended Data Fig. 4 — Unprocessed western blots. [file 41556_2023_1096_MOESM25_ESM.pdf]

Related to **Extended Data Figure 5C**

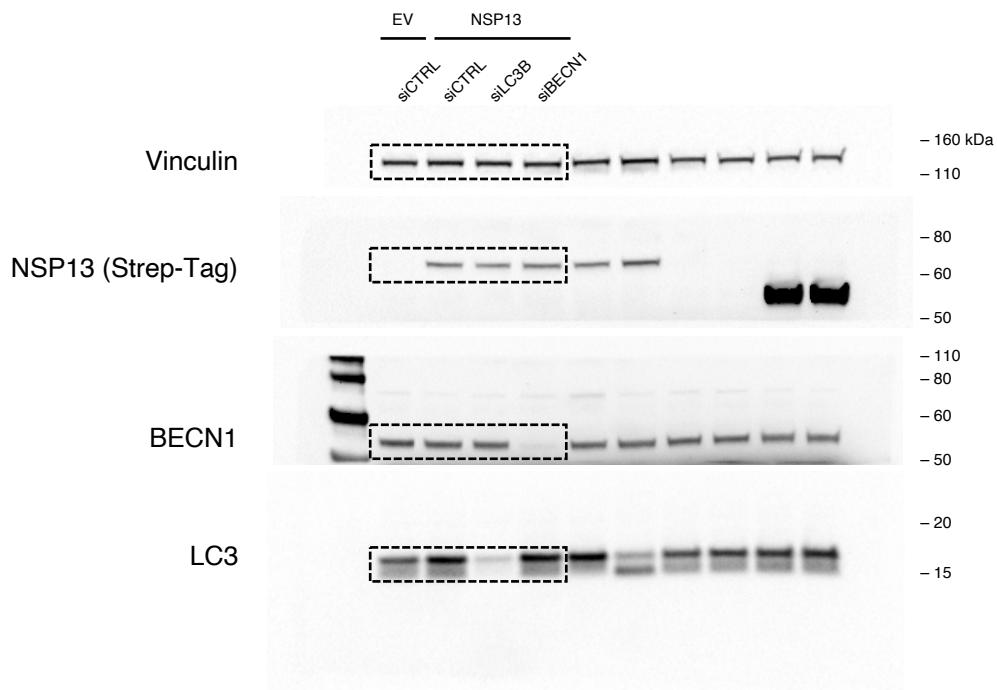

Supplement: Source Data Extended Data Fig. 5 — Unprocessed western blots. [file 41556_2023_1096_MOESM27_ESM.pdf]
