# Supplementary material for: SARS-CoV-2 infection induces DNA damage, through CHK1 degradation and impaired 53BP1 recruitment, and cellular senescence
Source: Nat Cell Biol. 2023 Mar 9;25(4):550–64. doi: 10.1038/s41556-023-01096-x (PMC10104783; doi:10.1038/s41556-023-01096-x)

Related to **Extended Data Figure 1A**

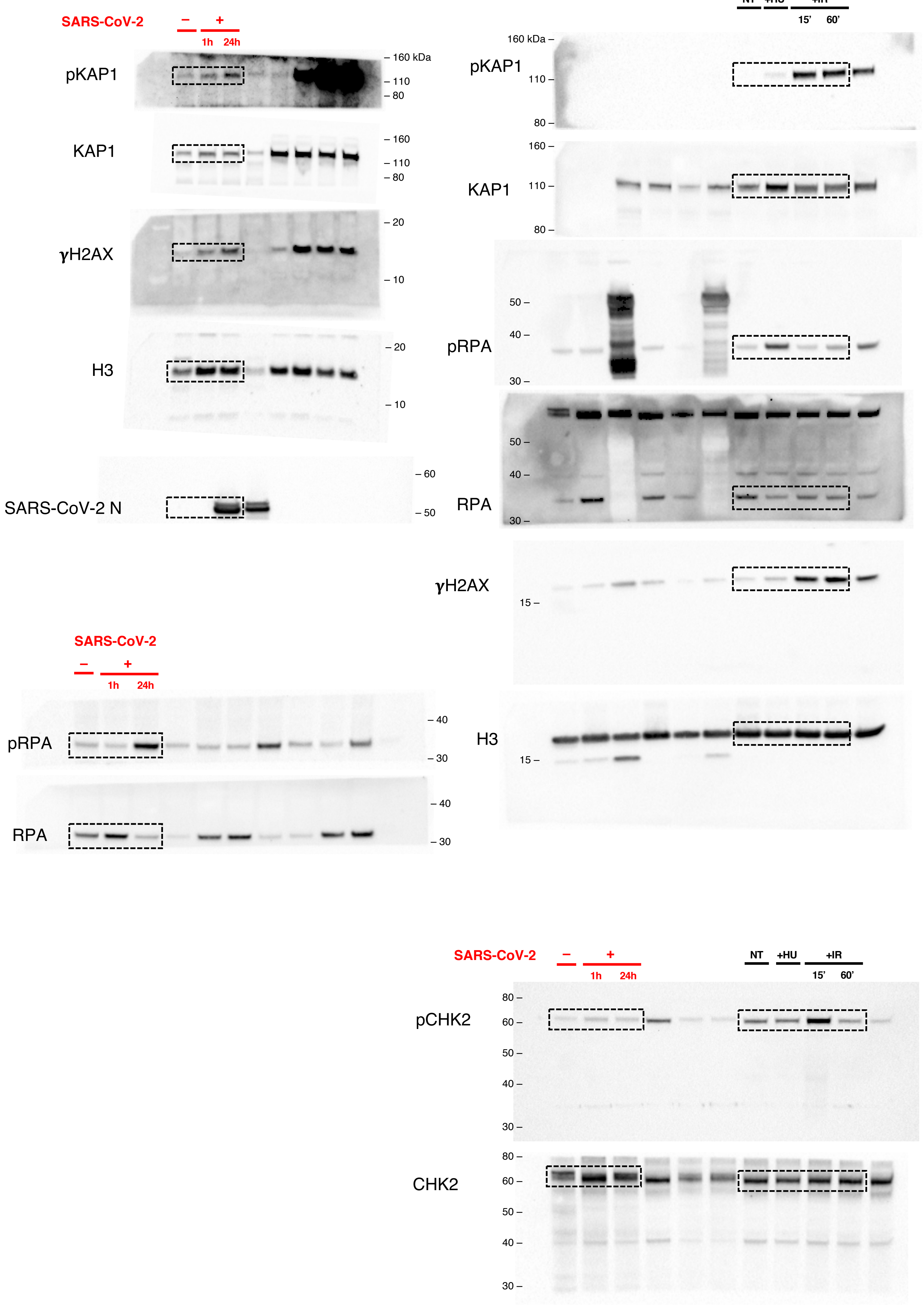

Related to **Extended Data Figure 1I**

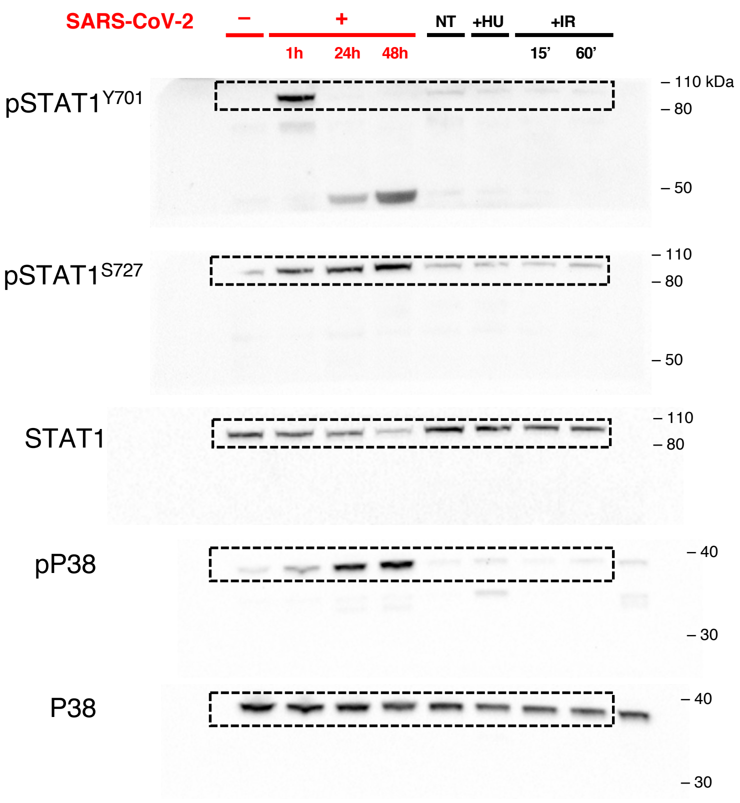

Related to **Extended Data Figure 1K**

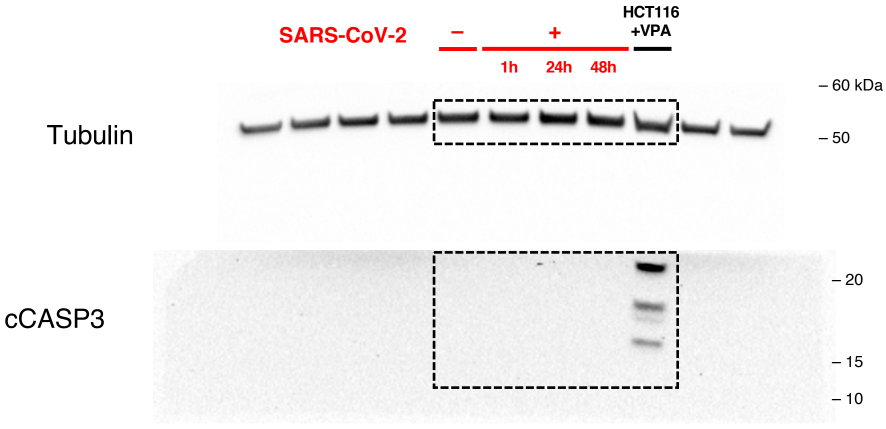

Supplement: Source Data Extended Data Fig. 1 — Unprocessed western blots. [file 41556_2023_1096_MOESM19_ESM.pdf]
